# Recent Applications of Metabolomics Toward Cyanobacteria

**DOI:** 10.3390/metabo3010072

**Published:** 2013-02-04

**Authors:** Doreen Schwarz, Isabel Orf, Joachim Kopka, Martin Hagemann

**Affiliations:** 1 Institut Biowissenschaften, Pflanzenphysiologie, Universität Rostock, Albert-Einstein-Str. 3, D-18059 Rostock, Germany; E-Mail: doreen.schwarz@uni-rostock.de; 2 Max-Planck-Institut für Molekulare Pflanzenphysiologie, Am Mühlenberg 1, 14476 Golm, Germany; E-Mails: orf@mpimp-golm.mpg.de (I.O.); kopka@mpimp-golm.mpg.de (J.K.)

**Keywords:** inorganic carbon, glucose feeding, modeling, mutant, primary metabolism, stable isotope labeling, synechocystis

## Abstract

Our knowledge on cyanobacterial molecular biology increased tremendously by the application of the “omics” techniques. Only recently, metabolomics was applied systematically to model cyanobacteria. Metabolomics, the quantitative estimation of ideally the complete set of cellular metabolites, is particularly well suited to mirror cellular metabolism and its flexibility under diverse conditions. Traditionally, small sets of metabolites are quantified in targeted metabolome approaches. The development of separation technologies coupled to mass-spectroscopy- or nuclear-magnetic-resonance-based identification of low molecular mass molecules presently allows the profiling of hundreds of metabolites of diverse chemical nature. Metabolome analysis was applied to characterize changes in the cyanobacterial primary metabolism under diverse environmental conditions or in defined mutants. The resulting lists of metabolites and their steady state concentrations in combination with transcriptomics can be used in system biology approaches. The application of stable isotopes in fluxomics, *i.e.* the quantitative estimation of carbon and nitrogen fluxes through the biochemical network, has only rarely been applied to cyanobacteria, but particularly this technique will allow the making of kinetic models of cyanobacterial systems. The further application of metabolomics in the concert of other “omics” technologies will not only broaden our knowledge, but will also certainly strengthen the base for the biotechnological application of cyanobacteria.

## 1. Introduction

The term “metabolome” was introduced to the scientific literature by Oliver *et al.* [1]. Metabolome comprises the complete set of small molecules or intermediates of cellular metabolism of an organism, organ, or cell, which is obtained via metabolomics, a technological platform of the “omic” toolbox for systems analysis. Metabolomics complements genomics, transcriptomics and proteomics at the metabolite level [2,3]. Naturally, the metabolome is determined by the coding capacity of the genome, and it is influenced by changes in the transcriptome and proteome but also by the direct or posttranslational impact of the chemical and nutritional environment of a cell on enzymatic and transport activities. In comparison to proteomics and transcriptomics, metabolomic analyses represent the level most closely related to the physiology of an organism, organ, or cell [4,5,6,7]. Like the other “omics” technologies, metabolomics became available due to the dramatic improvements of methods for metabolite analysis, mainly by the coupling of high throughput separation techniques via capillary electrophoresis (CE), gas or liquid chromatography (GC or LC) with new compound identification/quantification tools based on mass spectroscopy (MS) or nuclear magnetic resonance spectroscopy (NMR). These technologies enabled comprehensive and standardized metabolite analyses of complex biological samples. Like other “omics” technologies, the aim of metabolomics is the acquisition of large and comprehensive data sets, here ideally the complete set of cellular metabolites in relative or absolute quantitative terms. This aim is still difficult to achieve. The identification and quantification of all cellular metabolites through the fragment pattern via MS is a grand challenge due to the complex composition of the metabolome by highly diverse chemical molecules and their wide range of concentrations. Therefore, the optimization of the experimental procedure, the analytical technologies, and the data evaluation are major ongoing efforts in current and future metabolome research [8,9]. Despite the so far rather low coverage of state-of-the-art metabolome profiles compared to the close to comprehensive transcriptomic profiles, metabolomics is more directly connected to the physiology of organisms than all other “omics” technologies. Metabolic activities are often regulated not only by the amount of an enzyme but its activity state determines the metabolic activity. Especially the combination of steady state metabolomics with flux analysis using stable isotopes and their evaluation by mathematical modeling, *i.e.* current systems biology attempts, will allow a much better and comprehensive understanding of cellular metabolic networks and their flexibility under diverse conditions in a wide variety of organisms.

Here, we will review metabolomic approaches to understand cyanobacterial metabolism. The phylum cyanobacteria comprises photoautotrophic prokaryotes of the domain Bacteria. Ancient cyanobacteria invented oxygenic photosynthesis about three billion years ago, which was later conveyed by an endosymbiotic event into eukaryotes giving rise to the plastids [10]. Therefore, the photosynthetic metabolism in all phototrophic eukaryotes is phylogenetically related to that of cyanobacteria. Additional to photosynthesis, the cyanobacterial ancestor also contributed many more biochemical activities in present-day land plants. It has been estimated that approximately 18% of the protein coding genes of *Arabidopsis thaliana* were obtained via the cyanobacterial endosymbiont [11]. The close relationship of eukaryotic photosynthesis to that of cyanobacteria explains why cyanobacteria have been used for decades as a model to analyze plant functions, particularly related to photosynthesis.

Besides easy cultivation, the availability of genetic tools for cyanobacterial model strains supported the analysis of biochemical features. In 1996, the first genome of the most widely used cyanobacterial model strain, *Synechocystis* sp. PCC 6803 (hereafter *Synechocystis* 6803), was published [12]. Starting with this first cyanobacterial “omics” paper, genomics applied to cyanobacteria is still an expanding field. Meanwhile, more than 100 complete cyanobacterial genome sequences are available in public databases (e.g., CyanoBase: http://genome.kazusa.or.jp/cyanobase) [13]. Based on the available genome sequences, transcriptomic studies first applying the microarray technology were published with cyanobacteria (e.g. [14,15]). The *Synechocystis* 6803 microarray data are summarized in a specialized database (CyanoExpress 1.2: http://cyanoexpress.sysbiolab.eu/). Recently, the high throughput sequencing of cDNAs (RNAseq) was introduced to study cyanobacterial gene expression. This technique quantifies mRNA abundances as number of sequence reads and also allows the quantitative detection of hundreds of small, non-coding RNAs [16,17,18]. Transcriptome analyses soon became combined by proteomic studies initially using gel-based and later also gel-free high-throughput systems (e.g., [19,20]). Compared to genomics, transcriptomics and proteomics, the application of metabolomics to cyanobacteria started relatively late. While this technology was applied to land plants around the year 2000 [2,5], first examples for metabolomics with cyanobacteria appeared in the literature almost 10 years later with exception of the pioneering work by Yang and coworkers [21,22,23].

According to Fiehn, different metabolomic approaches can be distinguished: (i) metabolite target analysis, (ii) metabolite profiling, (iii) metabolite fingerprinting, (vi) metabolite footprinting, and (v) flux analysis [3]. The metabolite target analysis examines quantitatively specific metabolites or a few preselected metabolites, which are usually linked by an interest in common reactions or pathways [24]. Metabolite profiling investigates the amount of a group of known metabolites, which can be analyzed in parallel with a common analytical technology [2,5]. Metabolic fingerprinting aims for non-targeted, if possible non-biased, high-throughput quantification of intracellular metabolite pools (including still unknown metabolites) or in other terms the endo-metabolome. In contrast, metabolic footprinting analyzes the content of metabolites excreted from cells, the exo-metabolome [25,26]. Finally, the flux analysis applies stable isotopes such as ^13^C, ^15^N, ^18^O or classically radioactive isotopes to reveal the pathways in an organism or cell. High-throughput analyses of metabolite fluxes are summarized under the term fluxomics [27].

**Figure 1:**
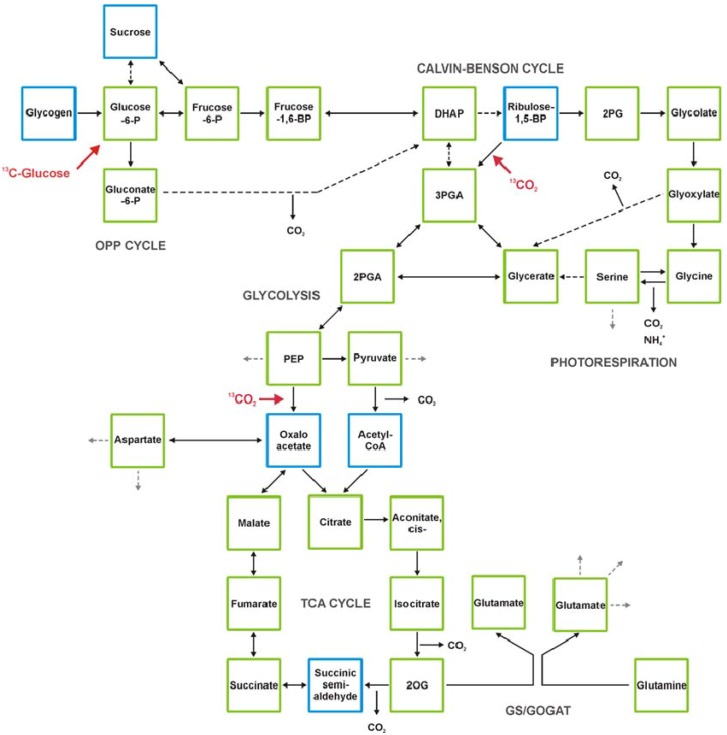
The primary metabolism of cyanobacteria and major entry points for inorganic carbon or glucose as organic carbon source (shown in red). Cyanobacteria such as *Synechocystis* 6803 fix CO_2_ mostly via the Calvin-Benson cycle, which is linked to the photorespiratory 2PG cycle. Organic carbon is exported to the sugar metabolism and eventually stored as glycogen, which is used as carbon source during the night via the oxidative pentose phosphate (OPP) cycle and finally respiration. Organic carbon also can be channeled into glycolysis and tricarboxylic acid (TCA) cycle mostly to produce carbon skeletons for biosynthetic purposes. The molecule 2-oxoglutarate (2OG) is taken as precursor for ammonium assimilation via glutamine-synthetase/glutamine-oxoglutarat-aminotransferase (GS-GOGAT) and represents the main link to nitrogen metabolism. Green squares label metabolites detected by gas-chromatography-mass-spectrometry (GC-MS) [22,51]. Blue squares show as for now non-detected metabolites by GC-MS. Dihydroxyacetone phosphate (DHAP), 2-phosphoglycolate (2PG), 3-phosphoglycerate (3PGA), 2-phosphoglycerate (2PGA), Phosphoenolpyruvate (PEP), 2-oxoglutarate (2OG).

The current state of technical developments and examples for the application of metabolomics to cyanobacteria will be summarized and discussed here. The use of metabolomics to understand cyanobacterial metabolism (Figure 1) will not only fill gaps in our knowledge on the metabolism of photoautotrophic cells. Cyanobacteria like microalgae are emerging candidates for the production of green energy [28]. To generate efficient cyanobacterial production strains, metabolomics will be a key technology to guide biotechnological approaches and strain design [29,30].

## 2. Technical Development towards Comprehensive Analysis of the Cyanobacterial Metabolome

Among the current “omics” technologies for biological systems analyses, metabolomics is perhaps the most generic. DNA, RNA and protein sequences are dependent on the organism of origin. In contrast, the origin of metabolites cannot directly be traced to the organism of origin. This holds true per definition for primary metabolites but is also applicable to many secondary metabolites. As a consequence, most aspects of the current metabolomic technology development are generic. We refer the reader to the wealth of previous reviews on metabolomics specifically its early emergence and application to plants [3,31,32,33,34,35,36,37]. One important aim of the metabolomics field is the continuous improvement of technologies [8,9]. This process includes efforts for improved data quality as well as analytical tools for increased sample throughput. Progress has been made in standardized metabolomic reporting and comprehensive documentation of experimental designs. Such efforts will serve to improve the notoriously difficult exact reproduction of physiological experimentation particularly with photosynthetic organisms. The full extent of metabolomic primary data and the increasing amount of accompanying metadata can only be handled efficiently using databases such as the Golm Metabolome Database (http://gmd.mpimp-golm.mpg.de/), which was developed for compounds in complex extracts from plant material [38,39,40,41]. The existing metabolomic databases are currently in the process of becoming interlinked, as has recently been envisioned by the MetaboLights initiative of the European Bioinformatics Institute (EBI) [42].

### 2.1. Sampling and Metabolic Inactivation

Adequate sampling technologies are crucial for reliable metabolomic snapshots of biological systems and are largely independent of the subsequently applied analytical technology. The means of inactivating cellular metabolic activities determines how accurate the *in situ* metabolome of living cells is preserved within subsequently generated metabolite profiles. This task is challenging in the case of photoautotrophic microorganisms such as cyanobacteria. The flux through the Calvin-Benson cycle and the associated photorespiration responds rapidly to changes of illumination or other environmental factors. As a consequence, metabolic inactivation of photoautotrophic metabolism must be as rapid as possible and attempt to maintain constant illumination and environment throughout sampling. Cyanobacteria are typically grown in liquid culture both for the purpose of laboratory experiments and for biotechnological production. Some cyanobacteria, if not all, are known to leave a metabolic footprint in culture media by release and uptake of metabolites [43]. As a consequence, modern metabolomic sampling technologies must separate suspended cells from the surrounding liquid medium to allow the acquisition of distinct metabolic profiles of the endo- compared to the exo-metabolome.

Calvin and Benson came up with an elegant solution in their groundbreaking work on the unicellular alga, *Chlorella*, which culminated in the discovery of the first photosynthetic assimilation product 3-phosphoglycerate (3PGA) [44]. Algal cells from the photoreactor were rapidly mixed with alcohol via a valve outlet guaranteeing rapid metabolic inactivation. Unfortunately, this strategy does not allow the differentiation between the endo- and exo-metabolome. A slight modification of the sampling procedure was developed for the analysis of intracellular metabolites from yeast suspension cultures, which are rapidly frozen in super-cooled, but still liquid methanol:water (70:30, v/v) mixtures. The metabolically inactivated cells can then be harvested by centrifugation. Cold methanol quenching was applied to cyanobacteria [45]. However, it also was shown that this quenching method reproducibly lead to losses of certain amino and carboxylic acids from cyanobacterial cells [46]. The same authors reported that quick centrifugation without quenching and washing followed by snap-freezing of the decanted pellet in liquid nitrogen resulted in reproducible metabolic snapshots. However, this technology can only be applied to heterotrophic growth conditions, as the cells are kept in the dark during centrifugation prior to metabolic inactivation.

An efficient solution of the combined separation and inactivation problem is fast filtration [22,46,47]. Fast filtration can be performed using glass vacuum filtration devices with controlled temperature and illumination. The filtration process can be as fast as 10 seconds [46]. During filtration, the cells are still illuminated and surrounded by liquid medium. After removal of the liquid, the filter and adherent cells are instantaneously frozen in liquid nitrogen and stored until further processing. Fast filtration is not only highly repeatable but can be multi-parallelized for the modern demands of medium- to high-throughput system analyses.

### 2.2. Technologies for the Profiling of the Cyanobacterial Metabolome

Metabolomics efficiently uses multiple analytical technologies, mostly hyphenated technologies that combine high-resolution separation with selective means of detection. Ideally, multiple technologies are combined to extend the - as a rule - limited metabolome coverage of single technologies and to independently verify the analytical results of overlapping metabolites. The advantages and disadvantages of different technological approaches have recently been reviewed [37]. Criteria, which are typically considered, are the range of covered metabolites, the selectivity of separation and detection, the means to unambiguously annotate known metabolites, and the potential to elucidate the nature of newly discovered but unknown metabolites.

The separation technologies currently applied for the analysis of the cyanobacterial metabolome are capillary electrophoresis (CE), liquid chromatography (LC), and gas chromatography (GC). CE is well suited for the separation of charged metabolites from primary metabolism, such as organic acids, phosphates and nucleotides [23]. The limitation to charged metabolites does not apply to GC and LC. Ultra (high) performance liquid chromatography (UPLC) is typically applied because UPLC achieves enhanced compound separation compared to conventional LC setups [48]. LC methods were also applied to separate polar compounds from cyanobacteria using reversed or normal stationary phases [49]. LC methods for broad-coverage metabolome analysis are frequently limited by separation problems of low molecular mass primary metabolites and are thus often tuned to the analysis of large primary metabolites, secondary metabolites or complex lipids. A widely applied technology and among the first applied to cyanobacteria is GC-MS profiling [22]. GC analysis initially applies to volatile compounds. For metabolomics, the coverage of GC-MS has been extended by chemical derivatization of polar metabolites, such as sugars, amino acids, organic acids, and phosphates into volatile products. This technology allows the analysis of a wide range of chemical metabolite classes in a single run, which was found to be especially convenient for the profiling of primary metabolism including uncharged primary metabolites and small secondary products.

The means of metabolite annotation are mostly dependent on the type of applied mass spectrometric (MS) technology. CE and LC are typically hyphenated to soft ionization MS technologies, such as electrospray (ESI-MS) or atmospheric pressure chemical ionization (APCI-MS), which typically maintain the molecular ion of a given metabolite. These ionizations are linked to MS systems that provide high mass resolution and accuracy. The recorded exact mass of a metabolite is used as the main criterion for metabolite annotation. Chemical isomers cannot be distinguished, because they have identical molecular formulas and consequently identical molecular masses. In contrast, GC is typically hyphenated to electron impact ionization (EI-MS), which generates highly repeatable and complex patterns of fragments that are suitable for automated matching procedures to mass spectral libraries.

MS provides data that support the structural elucidation of novel metabolites, but the gold standard for this purpose is nuclear magnetic resonance spectroscopy (NMR). ^1^H-NMR is frequently used for metabolome analysis without prior metabolite separation. ^1^H-NMR is less sensitive than the previously mentioned hyphenated technologies. The essential asset of ^1^H-NMR together with other NMR technologies is the access to structural information that reveal the position of atoms within novel compounds and the comparative ease of standardization for the absolute quantification of pool sizes. A method combining MS and NMR detection was developed and applied for the analysis of cyanobacterial extracts [50].

In the following, we will highlight aspects, which apply to cyanobacterial metabolomics with a focus on GC-MS-based metabolite profiling technology [22,47,51].

### 2.3. Standardized Data Processing and Specific Data Visualization

**Figure 2:**
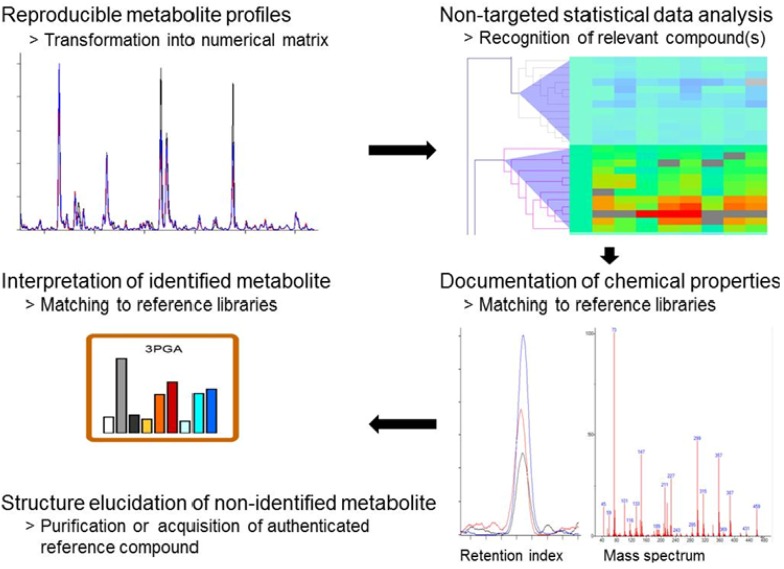
The essential workflow of non-targeted metabolite profiling. Non-targeted metabolite profiling generates reproducible and standardized metabolite profiles of biological samples. These profiles are transformed into numerical data matrices using automated data processing methods. Statistical data analyses identify compounds or groups of compounds, which are relevant for the investigated phenomenon. The chemical properties of the compounds of interest, in the case of GC-MS-based profiling, the retention index and the mass spectrum of a compound are matched to reference libraries such as the Golm Metabolome Database (http://gmd.mpimp-golm.mpg.de/). A positive matching result of a metabolite is prerequisite for the physiological interpretation, which is ideally done in context with metabolic pathways. A compound, which cannot be matched to reference information can be documented and referred to via the database entry of its chemical properties until final structural elucidation.

The general analytical procedure of the GC-MS-based profiling of primary metabolism has previously been described in detail [52,53]. The application of GC-MS to cyanobacteria generates highly complex and in the hands of experts highly reproducible chromatograms (Figure 2). The first step of standardized metabolomic data analysis is the pre-processing of chromatogram files into numerical data matrices that can be used for statistical data analysis. As typical metabolome profiling experiments comprise hundreds of compounds and also hundreds of chromatograms, metabolomic experiments are evaluated by statistical tools such as TagFinder [54,55]. The final result of metabolomic data pre-processing is a numerical table of corrected, so-called normalized responses, ready for statistical data analysis and visualization. It is important to note that non-targeted data pre-processing maintains information on all observed compounds defined by a mass and retention index (RI).

The final task of standardized data processing is to link observed compound mass and RI information to known metabolites. This annotation process is achieved by a matching process that uses databases such as the Golm Metabolome Database of mass spectral and RI information obtained from authenticated reference substances [40,41]. The annotation process can be supported by software [54,55], but should remain under manual supervision and must be augmented by verification through specific standard addition experiments to each newly investigated biological object. Due to these problems, accurate compound annotation is currently the bottleneck that limits the speed of GC-MS-based metabolite profiling studies. In the case of *Synechocystis* 6803, GC-MS-based profiling covers most of central metabolism (see Figure 1), including phosphorylated metabolites such as the products of ribulose-1,5-bisphosphate carboxylase/oxygenase (RubisCO), 3PGA and 2-phosphoglycolate (2PG), organic acid intermediates of the tricarboxylic acid (TCA) pathway, sugars, most amino acids, and other N-containing metabolites. An ideal resource for the interpretation of metabolomic data from cyanobacteria is the mapping of measured metabolite pools to the comprehensive inventory of pathways and enzymes reactions taken from full genome inventories [56]. Mapping of metabolome data serves the need to reduce and to simplify the complexity of “omics” data for the purpose of human interpretation.

**Figure 3:**
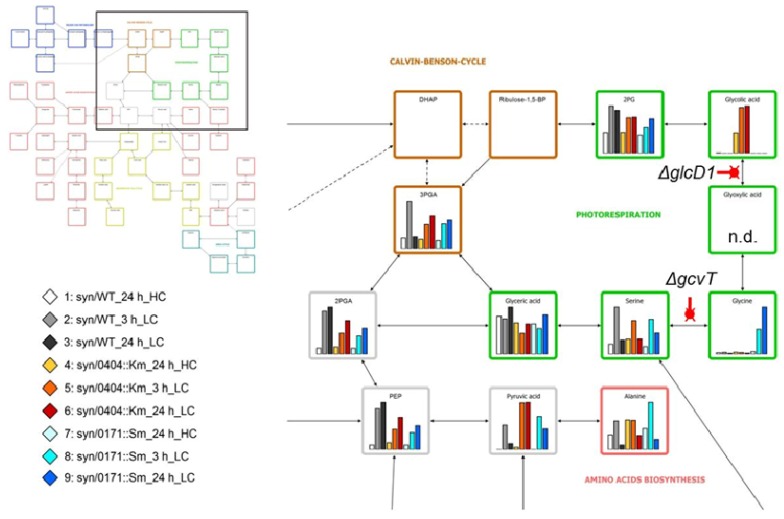
Mapping of metabolite profiles from the glycolate dehydrogenase 1 (Δ*glcD1*) (*sll0404*) and the glycine decarboxylase (Δ*gcvT*) (*sll0171*) mutants to primary metabolism of *Synechocystis* 6803 wild type. The pathway mapping was customized to the current coverage of GC-MS profiles of primary metabolism. The mapping visualizes the specific metabolic precursor accumulation phenotypes of the photorespiratory mutants defective in glycolate dehydrogenase 1 (Δ*glcD1*) and in the T-protein subunit of glycine decarboxylase (Δ*gcvT*), when shifted from high (5%) to low (0.035%) CO_2_ availability. Data were taken from the supplement of Eisenhut *et al.* [22]; n.d. (not detected). The top left insert shows the pathway overview and position of the zoom-in on photorespiration. The bottom left insert describes the color-coding of the bar diagrams. The display was partially automated using the VANTED software tool [58].

For the purpose of demonstration we choose here the data of Eisenhut *et al.* [22], which demonstrate strong precursor accumulation in two photorespiratory mutants, one defective in a glycolate dehydrogenase (Δ*glcD*) and the other in the T-protein subunit of glycine decarboxylase (Δ*gcvT*). The accumulation of glycolate and glycine, respectively, are linked to the changes in many other metabolites. But only the mapping of data onto the photorespiratory pathway reveals the context of precursor accumulations (Figure 3). Mapping tools, which allow visualization of metabolome data to full, partial, or simplified pathways are available for customized visual data interpretation [57,58,59].

### 2.4. Discovery of Still Unknown Metabolites

Metabolite profiles contain a vast amount of mass signals, which can currently not be attributed to known metabolites and are considered to represent a large number of still non-identified metabolites. The structural elucidation of all compounds that are observable by novel profiling technologies can be considered to represent the grand challenge of the metabolomics field [34,60]. In most studies, these non-identified compounds are disregarded. However, non-targeted metabolomics and specifically GC-MS-based profiling can also be used for the discovery of novel metabolites and not yet annotated pathways, which might be highly relevant to any investigated physiological phenomenon. Typically, a relevant but unknown metabolite is discovered by non-targeted statistical data analysis. The reference mass spectra and RI information of such novel compounds of interest can be archived, processed, and integrated in databases such as the Golm Metabolome Database prior to their full structural elucidation, which may take considerable time and effort (Figure 2). Thus still non-identified but archived metabolites, sometimes called the “known unknowns”, can be included into studies as marker substances relevant for defined physiological conditions. 

### 2.5. GC-MS-Based Metabolic Profiling Combined with Isotopic Tracing and Flux Analysis

**Figure 4:**
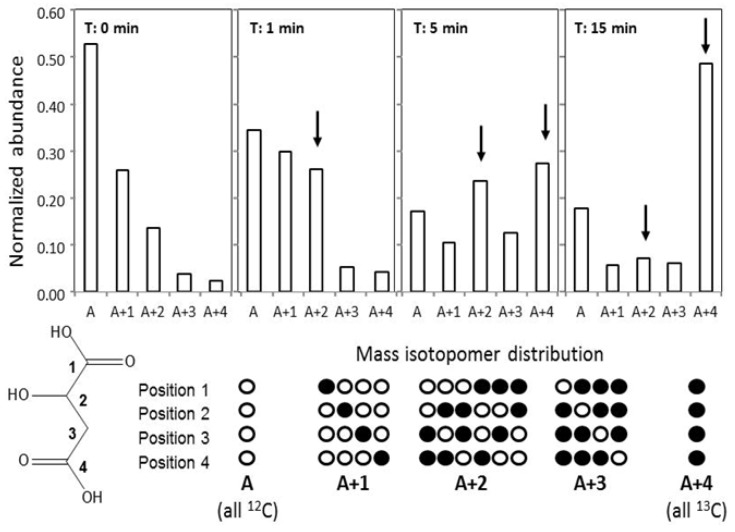
Mass isotopomer distributions of malate from a ^13^C-pulse experiment of *Synechocystis.* 6803 wild type pre-acclimated to 0.035% CO_2_. Mass isotopomer distributions are taken from a time course recorded at t_0_-t_15_ after a pulse with 2% (w/w) ^13^C-NaHCO_3_. Arrows indicate the preferential incorporation of two coupled ^13^C-carbon atoms, namely A+2 and A+4, into malate molecules. The mass isotopomer distribution at t_0_ reflects the naturally occurring isotopes of elements present in the malate molecule. The descriptor A represents the so-called monoisotopic mass of molecules, *i.e.* molecules that contain only ^12^C, the most abundant C-isotope, whereas A+1 to A+4 represent the mass of molecules, which contain one or in the case of malate up to four ^13^C-atoms. (All theoretically possible combinations of ^12^C and ^13^C atoms are given in the row below). Abundances are normalized to the sum of all mass isotopomers of malate. The malate ^13^C-enrichment reflects the non-random dilution of stable isotopes in biological systems. Note that the use of radioactive isotopes such as ^14^C does not directly provide information on the number of labeled carbon atoms in a molecule.

The first studies of metabolic flux analyses used radioactive isotopes. The detection of radioactive decay is highly sensitive. For this reason, very small amounts of labeled metabolites can be used, which do not significantly alter metabolite pool sizes. But radioactivity is toxic; therefore, radioactive isotopes have largely been replaced by stable isotopes. Stable isotopes usually need to be applied in higher amounts to meet the detection limits of current analytical technologies such as MS. The use of stable isotopes became possible, because MS provides information on so-called mass isotopomers. Isotopomers are mass-variants of a molecule, which arise from incorporation of naturally occurring or experimentally introduced elemental isotopes, such as ^13^C and ^15^N as compared to the in nature most abundant ^12^C and ^14^N isotopes. The incorporation of ^13^C or ^15^N causes mass shifts of +1 mass unit for each substituted atom in a molecule. For example, the natural isotopomer composition of malate from *Synechocystis* 6803 is shown in Figure 4. When stable isotopes are introduced into a biological system, their highest concentration or more exact “enrichment” is detected in those metabolite pools, which are immediately linked to the point of label incorporation. The enrichment pattern can be used to draw conclusions as to the connectivity of metabolite pools. When coupled to time course experiments, the flux of elements through a pathway from one metabolite pool to a defined connecting metabolite pool can be quantified. 

Finally, multi-parallel isotopomer measurements and available data on pool size concentrations can be fitted to mathematical models of metabolism. These models return the best fitting flux rates of all modeled enzymatic reactions. For example, the isotopomer composition of malate during a time course measured following a ^13^C-bicarbonate pulse demonstrated clearly that malate is predominantly synthesized by the joined incorporation of two C-atoms under the chosen conditions, likely by entry of acetate derived from phosphoenolpyruvate (PEP) into the TCA pathway of *Synechocystis* 6803 (Figure 4, discussed in more detail in Section 3.6). Fitting of such mass isotopomer data to mathematical models considering so-called carbon mappings will support intuitive interpretations and will provide estimates of the flux rates of enzymatic reactions [45], which can ultimately be compared between experimental conditions and genetically modified genotypes of *Synechocystis* 6803. The ground has been prepared for such studies by defining mass fragments and respective mass isotopomers of multiple primary metabolites, which are suitable for flux studies using GC-MS-based profiling allowing multi-parallel measurements of pool size and flux as shown with *Synechocystis* 6803 [47,61].

## 3. Metabolomics with Cyanobacteria

### 3.1. Early Approaches to Analyze Cyanobacterial Metabolites

Soon after the description of the Calvin-Benson cycle for photosynthetic carbon fixation [44], the path of carbon fixation was analyzed in cyanobacteria as ancestors of plant plastids. These early studies used ^14^C-labeled CO_2_ and followed its incorporation in different cellular metabolites during time. At that time, the metabolites were mostly separated by two-dimensional paper chromatography and identified via co-chromatography of authentic standard substances [62]. These techniques allowed the quantitative estimation of label accumulation in many phosphorylated intermediates of the primary cyanobacterial metabolism (see Figure 1). From these investigations it became clear that cyanobacteria like plastids use the C3-type of photosynthesis, however, PEP-carboxylase and/or carbamylphosphate synthase can contribute significantly to the inorganic carbon (C_i_) fixation at specific conditions [63]. Feeding cyanobacteria with ^14^C-labeled glucose and the identification of labeled intracellular metabolites also verified the dominating role of the oxidative pentose phosphate (OPP) cycle in sugar breakdown during dark periods, while the TCA cycle did not seem to contribute [64].

Subsequently, it was shown that the TCA cycle was incomplete among cyanobacteria. According to enzyme measurements and ^14^C-labelling with several cyanobacteria such as *Anacystis nidulans* (syn. *Synechococcus elongatus* PCC 6301), *Coccochloris peniocystis* or *Gloeocapsa alpicola*, the 2‑oxoglutarate (2OG) dehydrogenase complex is missing [65,66,67]. The currently available cyanobacterial genome sequences supported this view, because all of them did not contain genes for the 2OG dehydrogenase complex. Accordingly, it was assumed that the open TCA cycle only served to produce carbon skeletons for diverse biosynthetic pathways; particularly 2OG was used to produce glutamate via GS/GOGAT representing the main connecting point of the primary C- and N-metabolism. Correspondingly, 2OG was identified as a crucial signaling metabolite balancing the cyanobacterial C/N metabolism via the NtcA- and/or PII-based regulation of many genes and proteins involved in nitrogen assimilation [68,69]. Recently, the “open” TCA cycle could be closed (see Figure 1) by accessory enzymes among cyanobacteria [70]. First hints for a closed TCA cycle were obtained by Cooley *et al.*, who showed that labeled 2OG could quickly be converted into succinate in a *Synechocystis* 6803 mutant defective in fumarase [71]. But only in 2011, Zhang and Bryant identified two genes in the genome of *Synechococcus* sp. PCC 7002, which encode the 2OG carboligase and succinic semialdehyde dehydrogenase converting 2OG into succinate [70]. This study also applied present-day methods of metabolome analysis via GC-MS. Accordingly, the cyanobacterial TCA cycle is not open, but differs from the canonical one. It will be interesting to study, under which conditions or in which cyanobacterial strains the TCA cycle functions more as an opened or closed cycle. Similarly, the TCA cycle in plant leaf mitochondria usually functions as an open cycle delivering carbon skeletons for glutamate synthesis during the day, while it is more closed participating in respiratory functions during the night. This switch between an open and closed mode could fulfill regulatory tasks depending on the C/N state of cells [72].

### 3.2. Metabolic Target Analysis—Bioactive Compounds

Metabolic target analysis aims to analyze metabolites fulfilling common functions or being involved in defined target processes. Cyanobacteria are not only important models to analyze photosynthetic mechanisms but also produce diverse bioactive substances as secondary metabolites, which can have toxic effects on mammals and are of high concern in environmental and health policies [73,74]. Because of the high chemical variety of cyanobacterial bioactive compounds, such as cyclic peptides, polyketides, alkaloids, amino acid derivatives, and their high number of derivatives showing different toxic potential, the development of specialized analytical tools was and is a challenge. In the past, cyanobacterial toxins were often analyzed using bioassays with cell cultures and animals or antibody-based systems. Later on, chromatographic and NMR systems were developed allowing the targeted analysis of toxin classes [75]. Recently, high performance liquid chromatography (HPLC) or LC separation coupled to MS identification was also introduced to toxin analysis, which seemed to allow the quantification of diverse toxins by one method [76,77]. Using such tools, it is not only possible to monitor drinking water quality with increasing sensitivity; it also allows the MS-based identification of cyanobacterial toxins in environmental samples of marine cyanobacteria [78,79] or of lichens [80].

### 3.3. Metabolic Profiling—Fatty Acids

Metabolic profiling comprises approaches using metabolite spectra for the differentiation of species or phenotypes. The taxonomy of cyanobacteria is unclear [81]. Cyanobacteria are characterized by a high morphological and genetic diversity. According to the classical botanical approach, morphological properties were used to divide the cyanobacteria into five sections [82,83]. Based on the bacteriological taxonomy, cyanobacteria should be classified using a polyphasic approach, which takes many physiological and genotypic characters into consideration [81,84]. Today, the easiest and mostly used profiling technique applies the 16S rRNA sequence clustering. However, there are many attempts to include metabolite patterns as chemotaxonomic marker. The fatty acid composition was found to present a particular valuable marker in the polyphasic approach of microbial taxonomy [85]. Usually, the isolated fatty acids are converted to methyl esters and are analyzed by GC, which can be coupled to classical flame detection or more recently to MS-based detection. Libraries of fatty acid methyl esters exist allowing a bacterial classification according to this chemotaxonomic marker (e.g. http://www.midi-inc.com/pages/mis_libraries.html). Applying such a technology, the species composition of the cyanobacterial genera *Aphanizomenon*, *Microcystis*, *Nostoc*, and *Planktothrix* was analyzed [86,87]. The previously mentioned studies analyzed the whole-cell fatty acid composition, while Li *et al.* used minor fatty acid components like 2-hydroxy or 3-hydroxy fatty acids as chemotaxonomic markers for cyanobacterial strains [88].

Recently, mechanisms for cyanobacterial lipid and fatty acid biosynthesis as well as the corresponding product analysis received increased attention in the field of biofuel production. Fatty acids can be converted into biodiesel and other fuels of rather high energy density. The photoautotrophic life style of cyanobacteria will allow a CO_2_-neutral and regenerative fuel production [89,90].

### 3.4. Metabolic Fingerprinting

Metabolic fingerprinting is a rapid, untargeted and high-throughput method for the quantitative determination of the (whole) cellular metabolite spectrum, the metabolome. This technology is currently mostly used to globally characterize metabolic changes under different cultivation (environmental) conditions or genetic backgrounds. Among cyanobacteria, metabolic fingerprinting attempts used the separation of low molecular mass metabolites via CE, GC or LC usually coupled to MS detection and identification. These approaches revealed thousands of mass features mostly from unknown metabolites, which could be classified by statistical means (discussed above). For example, we compared the growth of *Synechocystis* 6803 under different CO_2_ concentrations in wild-type cells compared to mutants defective in photorespiratory enzymes [22]. The metabolic fingerprint revealed clear differences depending on the CO_2_ amount and/or on the genotype (Figure 3). Unfortunately, as in most cases, only part of the mass features could be assigned to chemical structures (usually between 50–100 metabolites). Therefore, many more differences in metabolites pattern have been obtained by the fingerprinting than can currently be tracked down to the cyanobacterial metabolism. Similar to proteomics, the combination of different metabolomics technologies is necessary to become more and more comprehensive.

Nevertheless, these fingerprints are extremely useful for the fast screening of alterations in the cyanobacterial metabolism under different growth conditions or in different genetic backgrounds. The growth and metabolic activities of cyanobacteria are determined and influenced by diverse environmental factors such as light, temperature, salinity, and the availability of different C or N sources [91,92]. Transcriptomics revealed increased or decreased expression of defined groups of genes for certain biochemical routes, *i.e.*, photorespiratory metabolism in *Synechocystis* 6803 [93,94]. These studies allowed to conclude that some metabolic routes are likely to be more or less active under certain conditions. However, not all changes in gene expression are directly translated into protein amounts, which also are often regulated at activity level. Therefore, the subsequent or combined application of metabolomics helps to clarify if anticipated transcriptional observations are transmitted into metabolic changes.

Among cyanobacteria, two key factors influencing growth have been investigated in much detail: The availability of carbon by shifts from high to low CO_2_ and/or by the addition of organic carbon sources such as glucose.

*CO_2_ availability:* Cyanobacteria use mainly C_i_ (CO_2_ and HCO_3_^−^) as primary carbon source. The availability of C_i_ determines the growth rate and the photosynthetic activity of cyanobacteria. Alterations in the gene expression pattern [93,94] and modifications of the proteome [95] were reported for *Synechocystis* 6803 in response to CO_2_ limitation. These studies revealed the complete set of low CO_2_-induced genes, among them the well-known transporters for inorganic carbon such as NDH-1, BCT1 and SbtA [96]. Additionally, many genes coding for enzymes of the primary C- and N-metabolism showed changed mRNA levels giving clear hints towards a coordinated change in the whole cell metabolism.

Subsequently, alterations of the metabolome in response to C_i_ limitation were investigated in *Synechocystis* 6803 [22]. The GC-MS-based analysis indicated that the Calvin-Benson cycle activity and glycogen storage decreased, whereas organic carbon was increasingly channeled into glycolysis (higher levels of dihydroxyacetone phosphate, 2-phosphoglycerate and PEP) and the TCA cycle (2OG, malate and fumarate increased). Moreover, photorespiratory metabolism became activated by low CO_2_. We could detect significant amounts of 2PG, the by-product of the RubisCO oxygenase reaction. These global metabolic changes were in accordance with changes of gene expression for some of the enzymes involved in the corresponding metabolic routes [93,94]. Much to our surprise, we found (at least transiently at the investigated time points of 3, 6, and 24 h after shifts from 5% to 0.038% CO_2_) an increase of the 2OG level while glutamate decreased. The 2OG level was supposed to decrease under lowered C_i_ due to the decreased C/N ratio. Previously, it has been shown that after a shift from a high to a low N-amount, *i.e.*, an increase in the C/N ratio, 2OG increases [97]. Therefore, the opposite behavior was assumed for a decreased C/N ratio. Recently, we found that another cyanobacterium, *Synechococcus elongatus* PCC 7942, showed very similar metabolic alterations including the (transient) increase of the 2OG level when shifted from high to low CO_2_ [51]. We interpret the increase of 2OG as a transitory lack of coordination or disequilibrium between the C- and N-metabolism during the first hours after CO_2_ starvation. To maintain growth, cells channel less organic carbon to storage and correspondingly more organic carbon to 2OG (and other TCA cycle intermediates) as precursors for amino acid and other biosynthetic processes. At the same time, the cells sharply decrease the uptake of nitrate and the ammonia assimilation via GS/GOGAT, because of the relative increase in N/C ratio after low CO_2_ shift. Thus, increased channeling of carbon toward the TCA cycle and reduced GS/GOGAT activity result in the accumulation of 2OG. This scenario is supported by the gene expression changes showing decreased expression of nitrate transporters and increased expression of proteins inhibiting GS activity in *Synechocystis* 6803 [94] as well as in *Synechococcus elongatus* PCC 7942 [51].

Thus, metabolomics reveal that shifts form high to low CO_2_ levels induce a coordinated change in the central C/N-metabolism. The PII protein is known as one of the central regulators for C/N balance in cyanobacterial cells [69]. The influence of PII on the shifts in the metabolomics fingerprint between high and low CO_2_ cells was investigated using the PII mutant of *Synechococcus elongatus* PCC 7942 [51]. Indeed, the metabolome and transcriptome of the PII mutant revealed characteristic alterations in comparison to the wild type. However, these alterations were mostly observed for N-containing metabolites and genes coding for enzymes involved in N-metabolism, *i.e.* the PII protein seems to be more responsible for the decrease in N-assimilation after shifts to low CO_2_ than for the changes specifically linked to C-metabolism [51].

*Glucose addition:* *Synechocystis* 6803 is one of the few cyanobacteria, which can take up and use external glucose as C-source. Therefore, photoautotrophic and photomixotrophic growth conditions can be directly compared with this strain. Transcriptomic [98,99] and proteomic [98,100] analyses of glucose effects on *Synechocystis* 6803 have been published during the last 10 years. Metabolomic fingerprints of glucose addition on the primary metabolism were published by Takahashi *et al.* and recently by Narainsamy *et al.* [23,48]. In agreement with the literature, the “omics” studies revealed that addition of 5 mM glucose under continuous illumination increased the steady state levels of many intermediates of the OPP cycle and glycolysis, whereas the intermediates of the Calvin-Benson cycle decreased. Moreover, the storage of carbon in sucrose and glycogen was stimulated by glucose, while the TCA cycle metabolites did not seem to be affected much [23,48]. Remarkably, the metabolomics approach also revealed that glucose addition to *Synechocystis* 6803 cells in light induced oxidative stress. Metabolites, which are characteristic for cells exposed to oxidative stress, were identified in the metabolome of cells grown under photoheterotrophic conditions [48].

*Metabolomics to characterize selected mutants:* The availability of metabolomics also made it possible to analyze the impact of genetic lesions in defined mutants on overall metabolism. For example, Miranda *et al.* aimed to characterize differences between wild-type and protease-mutant cells by a combined proteomics and metabolomics approach [101]. Because elevated temperatures were found to decrease the fitness of the mutant, both genotypes were compared in ambient and heat-stress conditions. The metabolic fingerprinting allowed a clear separation between wild-type and mutant cells, however, the temperature-induced changes overwrote the mutant–based differences.

Many glucose-sensitive *Synechocystis* 6803 mutants are known, but the basis of the glucose-sensitive phenotype is mostly unknown. The metabolic fingerprint of the glucose-sensitive *Synechocystis* 6803 mutant Δ*pmgA* (*sll1968*) indicated that the absence of PmgA (a protein of unknown function) resulted in failure to coordinate the carbon partition between the Calvin-Benson and OPP cycle. While wild-type cells increased the OPP cycle intermediates after glucose addition, cells of the mutant Δ*pmgA* activated the OPP to a much smaller extent. Probably, this metabolic imbalance leads to the glucose-sensitivity of the Δ*pmgA* mutant [23].

The primary carbon metabolism is regulated on different levels in the cyanobacterial cell. Alternative sigma factors seem to play an important role in the modification of key C- and N-assimilatory routes by changing the expression of genes for key enzymes under changed nutrient conditions. Recently Osani *et al.* have provided evidence that the SigE contributes to the differential expression of genes for enzymes involved in the carbon catabolism of *Synechocystis* 6803 [102]. A *Synechocystis* 6803 strain over-expressing the *sigE* was analyzed using transcriptomics, metabolomics and immuno-blotting to quantify the amount of key enzymes in the sugar catabolism. The results clearly indicate that SigE regulates the coordinated expression of sugar catabolism genes, especially those for the OPP cycle and TCA cycle enzymes. Deregulation of these enzymes changes the metabolic acclimation to altered growth conditions, for example, sensitivity towards glucose addition was found for the SigE overexpressing strain [102].

Glycogen represents the main carbon storage in cyanobacteria. Recently, *Synechocystis* 6803 mutants with impaired glycogen synthesis were constructed to analyze the role of this carbon storage during the acclimation to different growth conditions [103]. The mutation of glycogen accumulation indeed had severe effects on the viability of mutant cells. While the growth under continuous light was not affected much, the mutant became sensitive toward light/dark cycles, addition of glucose, and nitrogen starvation. Interestingly, glycogen mutants also showed the non-bleaching phenotype under N-starvation conditions, *i.e.* in contrast to wild-type cells, they showed retarded degradation of phycobilisomes and chlorophyll. Obviously, glycogen plays a more important role than just carbon storage in the cyanobacterial metabolism. The metabolome analyses revealed a lack of coordination of metabolism under fluctuating growth conditions when glycogen storage was abolished. Interestingly, the glycogen mutant selectively excreted high amounts of 2OG and pyruvate into the medium [103].

Mutants of *Synechocystis* 6803 also were used to analyze the contribution of specific enzymes toward the CO_2_-induced alterations in the primary C-metabolism. The metabolome in wild-type cells was compared to that of two photorespiratory mutants, Δ*glcD* and Δ*gcvT*. The mutant Δ*glcD* is defective in one of the two glycolate dehydrogenase genes, while Δ*gcvT* harbors a mutation in the T-protein of the glycine decarboxylase complex [22]. Both mutants showed the expected changes, *i.e.* Δ*glcD* accumulated glycolate and an increase of glycine was found in cells of Δ*gcvT* (see Figure 3). Interestingly, these photorespiratory intermediates accumulated already in mutant cells grown at high CO_2_, which showed that even under these conditions RubisCO oxygenation occurred, but the photorespiratory activity was sufficient to avoid the accumulation of these intermediates in wild-type cells. Under lower CO_2_ concentrations of ambient air, the two mutants accumulated much higher levels of the photorespiratory intermediates reflecting the increased photorespiratory activity based on higher RubisCO oxygenation. Additional to the expected changes in photorespiratory metabolites, the mutants, especially Δ*glcD*, already showed some features of the low CO_2_ metabolic fingerprint of wild-type cells under high CO_2_ conditions. These observations led to the conclusion that intermediates of the photorespiratory pathway, *i.e.* glycolate, might act as signal molecules for the C_i_ sensing among cyanobacteria [22]. This possibility was further investigated using another *Synechocystis* 6803 mutant, which cannot build carboxysomes, the central component of the C_i_-concentrating-mechanism. In cells of the mutant Δ*ccmM*, RubisCO is directly exposed to much higher oxygen levels and should show an increased oxygenase activity. Indeed, the metabolome of the mutant Δ*ccmM* showed accumulation of 2PG and other metabolites when sparked with 5% CO_2_, which were only found in wild-type cells when shifted to low CO_2_. However, the transcriptomics data obtained in parallel did not show any shift to the low CO_2_ gene expression pattern in high CO_2_-grown cells of Δ*ccmM*, which made a direct or exclusive role of 2PG as low C_i_ signal questionable [104].

As mentioned above, cyanobacteria are highly attractive cell factories for the production of green energy; one main trait is the increased hydrogen production. Under fermentative conditions, many cyanobacterial strains such as *Synechococcus* sp. PCC 7002 show the natural competence to produce hydrogen. However, the yield is low probably due to competing fermentative routes. Metabolic engineering was used in an attempt to improve the hydrogen production with this cyanobacterium [105]. To decrease the production of lactate, one of the main fermentation products of cyanobacteria, an *ldhA* mutant of *Synechococcus* sp. PCC 7002 was generated and characterized regarding changes in the endo- and exo-metabolome. As expected, the amount of lactate production decreased and more of the cellular reductants were used for alternative fermentation processes including hydrogen generation. Maximal 12% of the fermentative energy was converted into hydrogen in cells of the *ldhA* mutant [105]. This paper provides an example how metabolic engineering will become an attractive mean in the field of cyanobacterial biotechnology.

### 3.5. Metabolic Footprinting

Microorganisms interact permanently with their environment by uptake or release of metabolites. Metabolic footprinting is often used as a term for metabolome analyses of extracellular compounds in growth media. The accumulation of compounds in the surrounding medium can result from their release by exudation, *i.e.* export of substances via defined transporters or channels, or by (partial) cell lysis. As photoautotrophic prokaryotes, cyanobacteria are regarded to use inorganic nutrients and light for the production of organic cell material. However, there are many reports that cyanobacteria can also import and use organic substrates from the environment. A specific glucose transporter allows *Synechocystis* 6803 to grow with organic carbon instead of CO_2_ as C-source [106]. For oceanic *Prochlorococcus* spp. it also was shown that amino acids could serve as significant N-source [107]. Different transporters for neutral, basic and acidic amino acids as well as for compatible solutes such as trehalose, sucrose, glucosylglycerol, and glycine betaine have been identified [68,92]. However, the mutation of these transport systems resulted in the leakage of these compounds from the cells indicating that these transporters were mostly required to avoid their losses. There are also reports that natural populations of cyanobacteria release organic carbon, mostly as glycolate, as well as organic nitrogen to the environment under specific conditions. Cyanobacterial exudates then can be used by heterotrophic bacteria [108,109].

Nowadays, metabolic footprinting can be done in a high through put mode combining stable isotope labeling with modern analysis tools like LC-MS or GC-MS. Recently, Baran *et al.* performed the first comprehensive study to investigate the uptake and release of metabolites by axenic cells of *Synechococcus* sp. PCC 7002 [43]. The authors cultivated this cyanobacterium in different media (minimal and three media enriched with organic nutrients) and compared the time-dependent changes in media composition. An impressive large spectrum of organic compounds was found to be taken up by this cyanobacterial strain. Especially, when the minimal medium was enriched with low molecular compounds extracted from *Synechococcus* sp. PCC 7002, many of the cellular metabolites could be imported illustrating the capability of cyanobacteria to avoid or minimize the loss of valuable cellular compounds. The authors also showed that compounds, which were previously not reported as cyanobacterial metabolites such as histidine betaine or γ-glutamylphenylalanine, were not only found in the cell extracts but were also taken up by cells of *Synechococcus* sp. PCC 7002. Applying stable isotopes made it possible to show that for example adenine and glutamate were not only taken up but also actively incorporated into the cellular metabolism. These data allowed the conclusion that the uptake of diverse metabolites allows *Synechococcus* sp. PCC 7002 to benefit from the availability of organic nutrients, which are released into the medium by lysis of cells after stress, viral attacks, or predation [43].

### 3.6. Flux Analysis

The application of radioactive or, in our days, stable isotopes in labeling experiments allows following the carbon or nitrogen flux in an organism. Compared to metabolic fingerprinting, in which changes in the steady state levels of metabolites are detected, flux analysis allows the direct recognition whether or not the synthesis or breakdown of specific intermediates is affected.

Relatively early, Yang *et al.* used ^13^C-labeled glucose to investigate the metabolic reorganization in *Synechocystis* 6803 under heterotrophic and mixotrophic growth [21]. The study revealed the OPP pathway as the dominant path of glucose utilization under heterotrophic conditions. In contrast, under mixotrophic growth the CO_2_ fixation through the Calvin-Benson cycle exceeded glucose assimilation. *Synechocystis* 6803 did not only prefer CO_2_ assimilation via RubisCO under photoheterotrophic conditions, it also employed the PEP carboxylase to increase CO_2_ assimilation into the TCA cycle and the glycolysis. Based on these findings, Yang *et al.* postulated the functioning of a C4 pathway in cyanobacteria [21]. In an accompanying study, the effects of glucose addition on expression levels (protein and mRNA) of key enzymes for C-assimilation were analyzed [98,110]. This comprehensive analysis revealed that most changes in the carbon flow could not be explained by expression changes. Therefore, the authors concluded that the regulation of enzymatic activities via modified substrate concentrations and/or by light-induced redox changes was responsible for the observed alterations of the C-fluxes.

Recently, Huege *et al.* and Young *et al.* used ^13^C-bicarbonate pulse-labeling to define the major C_i_ fixation pathways in the cyanobacterium *Synechocystis* 6803 [45,47]. The enrichment of ^13^C in selected metabolites was followed up to 60 min after addition of ^13^C-label. The ^13^C enrichment kinetics showed that compounds closely linked to the Calvin-Benson cycle became completely saturated by ^13^C, while other pools such as these of sucrose or TCA cycle intermediates showed relatively slow ^13^C enrichments.

Our experiments revealed two major pathways for the C-assimilation in the light. The main CO_2_fixation was done by RubisCO and the organic carbon was channeled into (i) sucrose via glucose-6-phosphate and (ii) aspartate, malate, citrate and 2OG via PEP [47]. Low CO_2_-acclimated cells increased the second path considerably. Moreover, comparison of labeling kinetics of 3PGA *versus* 2PG was used to quantify carboxylation/oxygenation ratios. Already at high CO_2_ a low label of 2PG was found, which increased after shifts to low CO_2_ conditions amounting to maximal 4% oxygenation. Additional to RubisCO, carboxylation via PEP carboxylase was active, which showed increased contributions to the overall ^13^C-incorporation in the two photorespiratory mutants Δ*gcvT* and Δ*glcD* [47]. The manual inspection of malate labeling (see Figure 4) provided indications for the prevailing simultaneous addition of two ^13^C-atoms into malate over addition of a single ^13^C-atom by direct ^13^C-bicarbonate incorporation via PEP carboxylase. The simultaneous addition of two ^13^C-atoms can be explained by insertion of acetyl-CoA into the TCA pathway of *Synechocystis* 6803 and subsequent synthesis of malate from the intermediates of this path. If this assumption is true, the labeling pattern of malate could be used as an indicator to analyze the degree of an open *versus* a more closed TCA cycle under specific conditions. However, the relative contributions of PEP carboxylase and/or the TCA pathway and the exact route of malate synthesis still need to be modeled and explored in more detail.

Young *et al.* already combined stable isotope labeling and a model approach, *i.e.* they used the isotopically non-stationary metabolic flux analysis (INST-MFA) [45]. The MFA allowed the analysis of isotopomer patterns leading to a detailed as well as quantitative description of CO_2_ fixation in cells of *Synechocystis* 6803. Thus, the authors provided the first kinetic modeling of photoautotrophic growth directly based on measured ^13^C-fluxes, which was evaluated regarding potential targets of optimization of photosynthetic carbon fixation to support biotechnological applications of cyanobacteria [45]. This analysis quantified precisely the rates of all Calvin-Benson cycle reactions, therefore, the total CO_2_ fixation could be related to the incorporation of organic carbon into the biomass. The authors reported that about 25% of initially fixed CO_2_ was lost during photoautotrophic growth. The parallel investigation of the exo-metabolome revealed that the release of organic carbon into the medium did not play a significant role. Beside CO_2_ losses due to respiration, the OPP cycle was found to be a significant process leading to diminished carbon use efficiency. Initially, it was thought that the OPP cycle becomes inactivated in light-exposed cyanobacterial cells. At least under the experimental conditions used by Young *et al.*, the OPP pathway was still active in the light possibly to balance redox or precursor needs [45]. Moreover, significant amounts of CO_2_ were also released by the activity of the malic enzyme. However, the malate seemed to be mostly synthesized via C4-like carboxylation of PEP leading to oxaloacetate and then malate, making this pathway CO_2_ neutral. Most probably the combined action of PEP carboxylase and malic enzyme does not serve as CO_2_ concentration mechanism as in C4 plants but is rather responsible for an increased synthesis of pyruvate as precursor for amino acids or for other compounds. Photorespiration, *i.e.* carbon loss due to RubisCO oxygenase activity based 2PG production, was ruled out as a main pathway diminishing the efficiency of photoautotrophic growth of *Synechocystis* 6803. Under the CO_2_ and light conditions used by Young *et al.*, oxygenation accounted for only 0.5% of RubisCO carboxylation [45]. Thus, this study proposes possible targets for the metabolic engineering of cyanobacterial C-assimilation to minimize those “unwanted” reactions to improve growth and productivity of the cells.

The cyanobacterium *Cyanothece* sp. ATCC 51142, an N_2_-fixing unicellular strain, has recently received much attention as a potential producing host for hydrogen [111]. In order to improve the future biotechnological application of this strain, its primary C- and N-metabolism were analyzed using ^13^C-glycerol feeding. The effect of the organic carbon on primary metabolism and growth was analyzed in the presence of light and other growth conditions [112]. These analyses showed that organic carbon significantly reduced autotrophic CO_2_ fixation in *Cyanothece* ATCC 51142, *i.e.*, the cells grew mostly heterotrophically in the light when supplemented with organic carbon, while glucose addition had a much lower impact on *Synechocystis* 6803. Moreover, a detailed analysis of the isotopomer composition of amino acids allowed the discovery of a new isoleucine synthesis pathway among cyanobacteria [113]. Previously, it was assumed that cyanobacteria like most other bacteria synthesize isoleucine from threonine via 2-ketobutyrate. Because the enzyme for the threonine/2-ketobutyrate conversion was not annotated in the genome of *Cyanothece* sp. ATCC 51149, the authors searched for an alternative isoleucine synthesis. The isotopomer analysis of ^13^C-labeled isoleucine obtained after ^13^C-glycerol feeding indicated that this amino acid is synthesized by the citramalate pathway, in which acetyl-CoA and pyruvate are condensed to citramalate as precursor of 2-ketobutyrate, rather than using threonine as precursor. The isoleucine pathway deduced from the isoleucine labeling pattern was supported by the identification of the citramalate synthase gene in the genome of *Cyanothece* sp. ATCC 51142 [113]. Because similar proteins are encoded in some other cyanobacterial genomes, the isoleucine biosynthesis differs among cyanobacterial species.

## 4. Future Perspectives

Metabolomics, particularly in combination with the other “omic” tools such as transcriptomics or proteomics, represents a powerful tool for the investigation of basic physiological phenomena. Fiehn described metabolomics as the link between genotype and phenotype [3]. Clearly, the application of this technology to various aspects of basic or applied research with cyanobacteria will rise during the near future. As discussed here, the basic technology is available and can be used after appropriate standardization to specific model organisms.

One of the major challenges for metabolomics will be the future identification of hitherto unknown compounds. A broader coverage of metabolites will not only allow a deeper insight into the cyanobacterial metabolism, it also will certainly lead to the discovery of biochemical routes presently not assigned to the cyanobacterial kingdom. It is noteworthy that each cyanobacterial genome harbors up to 30% of genes for proteins with unknown function, even some enzymes for pathways of the primary C- and N-metabolism are virtually missing (e.g. phosphoserine pathway, [56]; isoleucine synthesis in *Cyanothece* sp. ATCC 51142, [113]). Metabolomics with mutant collections defective in genes for such unknown proteins will present a perfect tool for the functional annotation of these proteins. The accumulation of defined metabolites could provide hints for their biochemical roles. In many cases, related proteins are found in other organisms including land plants; therefore, the functional annotation of these proteins among cyanobacteria will close gaps in our general knowledge of photoautotrophic metabolism.

Besides metabolic fingerprinting, fluxomics using labeled precursors promise the best insights into the cyanobacterial metabolism and its regulation. As shown by Young *et al.*, the evaluation of such labeling experiments needs state of the art modeling approaches [45]. In the moment, most models are rather descriptive applying flux balance analysis [56]. These models allow metabolic network reconstruction and can be used to search for pathway gaps or to plan synthetic biology experiments for the production of biofuels. The challenge will be to develop kinetic models, which do not only allow the evaluation of labeling experiments, but also integrate data from corresponding experiments analyzing gene expression changes. Such a system level understanding of the cyanobacterial physiology promises to predict sensitive steps for the sustainable production of biomass, biofuel, or chemical feedstock based on solar energy and CO_2_ using a cyanobacterial cell factory as discussed recently by Nogales *et al.* [114].
